# Factors associated with *Chlamydia trachomatis* testing in a high school based screening and previously in clinical practice: a cross-sectional study in Norway

**DOI:** 10.1186/1471-2334-13-361

**Published:** 2013-08-01

**Authors:** Kirsten Gravningen, Gunnar Skov Simonsen, Anne-Sofie Furberg, Tom Wilsgaard

**Affiliations:** 1 Department of Microbiology and Infection Control, pb 56, University Hospital of North Norway, Tromsø N-9038, Norway; 2 Department of Community Medicine, Faculty of Health Sciences, University of Tromsø, Tromsø N-9037, Norway; 3 Department of Medical Biology, Research Group for Host-Microbe Interaction, Faculty of Health Sciences, University of Tromsø, Tromsø N-9037, Norway

**Keywords:** Chlamydia trachomatis, Testing, High school screening, Adolescents, Gender differences

## Abstract

**Background:**

High school based chlamydia screening has been shown to increase uptake and detect hidden infections among sexually active adolescents. Our study aimed to: i) examine the proportions of 15–20 year-olds tested in a high school based screening and previously in clinical practice, ii) determine chlamydia prevalence according to testing pattern, and iii) examine factors associated with testing in the two settings.

**Methods:**

A population based cross-sectional study was conducted in 5 high schools in Norway in 2009, using web-questionnaires and *Chlamydia trachomatis* PCR in first-void urine (800 girls/818 boys, mean age 17.2 years). Only sexually active participants at risk for chlamydia infections were included in the analyses. Crude and multivariable logistic regression models were applied with ‘clinic based testing’ and ‘school based screening’ as outcome variables.

**Results:**

56% of girls and 21% of boys reported previous clinic based testing. In the school based screening, 93% were tested with no gender difference. 42% of girls and 74% of boys were tested for the first time at school (‘school-only test’). Both girls with clinic based testing and girls with school-only test had high chlamydia prevalence (7.3% vs 7.2%). Boys with clinic based testing had twice the prevalence of those with school-only test (6.2% vs 3.0%, *p =* 0.01). Half of infections were detected in participants with school-only test. One-fifth were repeat infections. In multivariable analysis of girls and boys combined, female gender, older age, early sexual debut, no condom use at first and last intercourse, steady relationship, and higher number of lifetime partners increased the odds of clinic based testing. The odds of school based screening increased with male gender, academic affiliation, later sexual debut, condom use at first intercourse, and current urogenital symptoms in multivariable analysis.

**Conclusions:**

More than half the girls had been tested prior to the school based screening and had high prevalence independent of previous clinic based testing. School screening was mostly associated with factors unknown to increase chlamydia infection risk, while clinic based testing was associated with traditional risk factors. The unusually high and equal participation between genders and the detection of a large chlamydia reservoir confirms the value of school based screening suggesting this approach to be further explored in Norway.

## Background

Adolescents have a disproportionate burden of genital *Chlamydia trachomatis* infections, but may not feel at risk since most infections are asymptomatic and testing rates remain low [1-3]. Repeat chlamydia infections are common in adolescents [4]. According to Norwegian surveillance data, the female to male chlamydia test ratio has been more than 4 to 1 in age group 15–19 years with average positivity rates of 15% in girls and 18% in boys, respectively [2]. Accurate annual testing rates and rates of repeat testing in the Norwegian adolescent population cannot be estimated due to lack of unique personal identifiers. The major predominance of females is in line with chlamydia screening programmes in other high-income countries and may reflect gender differences in health seeking behaviour [5,6].

Norwegian health authorities recommend chlamydia testing in the presence of clinical symptoms, or if partner is infected, or in persons younger than 25 years if change in sexual partner [7]. Testing and treatment in these groups are free of charge. Test of cure is recommended 5–6 weeks after treatment. The majority of chlamydia testing among adolescents is done in general practice and in public youth clinics which are tailored to the needs of adolescents and are present in most municipalities. Youth clinics offer contraceptive counselling without parental consent and all services are free. Most high schools have a school nurse available part time providing general health services that only include limited chlamydia testing. Sexually transmitted infection (STI) clinics are available only in a few large towns.

Expansion of chlamydia testing from clinical practices to school based settings has been shown to increase uptake among adolescents, particularly in boys [8-12]. A number of extensive chlamydia screening programmes have been implemented in high schools in the US [9-11], but less so in Europe [8,13]. In 2009, we conducted a cross-sectional study on early sexual behaviour and chlamydia infection among high school students in Norway. Among the sexually active, chlamydia prevalence was 7.3% (95% confidence interval, CI, 5.3–9.7%) in girls and 3.9% (2.3–6.0) in boys with infections starting to be acquired soon after sexual initiation [14]. This study provided an opportunity to examine factors associated with chlamydia testing in a school based screening and previously in clinical practice, and to estimate the chlamydia reservoir in adolescents not seeking testing on their own. As school based chlamydia screening is not current policy in Norway, we assumed that previous testing had been done in clinical practice, ie ‘clinic based testing’.

The objectives of this paper were to; i) examine the proportion of adolescents aged 15–20 years in Norway tested in a high school based screening and previously in clinical practice, ii) determine chlamydia prevalence according to testing pattern, and iii) examine demographic and sexual behavioural characteristics associated with school based screening and previous clinic based testing.

## Methods

A detailed description of the study has been reported elsewhere [14]. In brief, a population based cross-sectional study was conducted in 5 public high schools in Finnmark county in Northern Norway in 2009 using a web-questionnaire and first-void urine (FVU) samples. All data were collected by the same experienced female doctor and nurse who consecutively visited a total of 123 classes using an identical approach. Written information about chlamydia infection, questionnaire items, and sampling procedures were handed out in class two weeks prior to data collection. Confidentiality regarding questionnaire data and chlamydia test results was assured both in the written information and later by oral repetition in each class. On the day of data collection, a web-questionnaire was emailed class-wise to each student including questions on demography, substance use, sexual behaviour, contraceptive use, current urogenital symptoms, and prior chlamydia testing and treatment. The teacher and study staff were present in class while participants filled in the questionnaire on their laptops. Directly thereafter, participants went on to the school toilets where they provided about 12 ml FVU samples under supervision of the study nurse. Samples were immediately refrigerated and delivered to the laboratory on the following day for *C. trachomatis* PCR testing (ProCt real-time PCR, ProCelo as, Tromsø, Norway). Test result notification time was 1–2 days. Participants testing positive were called on their cell phone by the nurse and given an appointment at the local youth clinic. Infections were treated with a single dose of 1 gram azithromycin orally.

**Figure 1:**
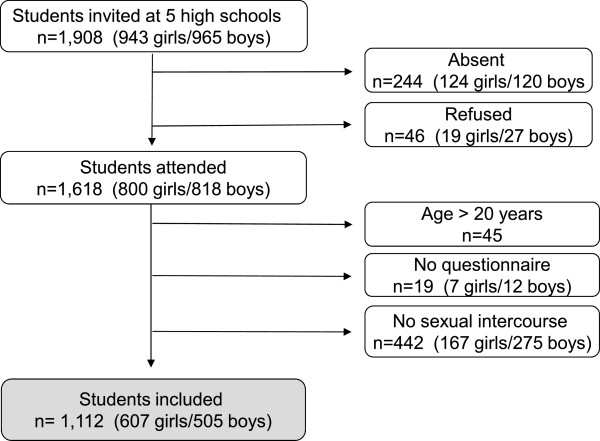
Study population.

Overall participation rate was 85% (1,618 of 1,908) (Figure 1). If only assessing students present at school, 2% (46 of 1,664) refused participation. 442 participants responding ‘no’ to: ‘Have you ever had sexual intercourse?’ were considered not to be at risk for chlamydia infection and were excluded from the analyses. All 442 had negative test results. 1,112 participants reporting sexual intercourse experience were considered to be at risk and were included. Mean age was 17.2 years (standard deviation, SD 1.0, median age 17.0 years).

The variable ‘high school study affiliation’ was defined as; 1) ‘academic’ , including students in the general academic studies programme, and 2) ‘vocational’ , including vocational school students. In Norway, academic and vocational classes frequently share facilities throughout high school.

Previous clinic based testing was assessed by; ‘Have you previously been tested for genital chlamydia infection?’ with response options: ‘Yes, once’ , ‘Yes, twice’ , ‘Yes, 3 times’ , ‘Yes ≥ 4 times’ , or ‘No’. Due to small groups, the variable ‘clinic based testing’ was dichotomised as yes/no. ‘School based screening’ included all participants that were screened in the high school study independent of clinic based testing. The subgroup ‘school-only test’ included participants with no previous clinic based testing that provided a urine sample in the school based screening.

### Statistical analysis

Descriptive characteristics were reported with means (SD) for continuous variables and with numbers (%) for categorical variables. The 95% CI for proportions were calculated using the exact binominal method. Crude and multivariable logistic regression models were applied with two dependent variables: 1) ‘clinic based testing’; yes/no, and 2) ‘school based screening’; yes/no. All analyses were performed separately for girls and boys and in both genders combined. Variables with *p* value < 0.25 in crude analysis were included in the multivariable models which were fitted using stepwise elimination. Age and gender (if applicable) were included regardless of significance. Collinearity was not a problem with variance inflation factor (VIF) < 2.5 for all variables. Gender interaction was assessed by including cross-product terms between each independent variable and gender. Statistically significant interaction terms were included in the final multivariable model. Model fit was assessed using Hosmer and Lemeshow goodness-of-fit test with 5 of 6 *p* values > 0.25. All statistical tests were two-sided using a 5% significance level and were performed in SPSS 19.0 (IBM Corp., New York, US).

### Ethics

Written informed parental consent was obtained for participants < 16 years. Participants ≥ 16 years gave their informed consent by filling in the web-based questionnaire. The study was approved by the Regional Committees for Medical and Health Research Ethics North Norway.

## Results

Clinic based chlamydia testing was reported by 56% of girls and 21% of boys (Table 1) with more girls than boys reporting multiple tests (61% vs 34%, *p <* 0.001). In the high school based screening, 93% of sexually active participants, 564 of 607 girls and 470 of 505 boys, were tested with no gender difference (Additional file 1). 42% of girls and 74% of boys were tested for the first time in the school based screening, i.e. school-only test.

**Table 1:** **Sosiodemographic and sexual behaviour characteristics - prevalence and crude odds ratios for *clinic based testing***

	**Girls**								**Boys**				**All participants**	
	**Clinic based testing**								**Clinic based testing**					**Interaction**
**Characteristic**	**N**	**n**	**(%)**	**OR**	**95% CI**	***p***^**1**^	**N**	**n**	**(%)**	**OR**	**95% CI**	***p***^**1**^	***p***^**1**^	***p***^**2**^
**Total**	607	338	(55.7)	NA			505	106	(21.0)	NA				
**Age**														
15-16	179	81	(45.3)	1.00		*< 0.001*	149	19	(12.8)	1.00		*0.001*	*< 0.001*	*0.80*
17	193	105	(54.4)	1.44	0.96–2.17		196	40	(20.4)	1.75	0.97–3.18			
18	177	104	(58.8)	1.72	1.13–2.62		114	28	(24.6)	2.23	1.17–4.24			*,*
19-20	58	48	(82.8)	5.81	2.77–12.20		46	19	(41.3)	4.81	2.25–10.29			
**Family and culture**														
Ethnicity							,							
Norwegian	433	232	(53.6)	1.00		*0.049*	353	56	(15.9)	1.00		*< 0.001*	*0.001*	*0.022*
Sami/Sami-Norwegian	131	85	(64.9)	1.60	1.07–2.40		115	37	(32.2)	2.52	1.55–4.08			
Other	43	21	(48.8)	0.83	0.44–1.55		36	13	(36.1)	3.00	1.43–6.27			
Residence in school year														
At home	380	196	(51.6)	1.00		*0.007*	314	55	(17.5)	1.00		*0.015*	*0.001*	*0.78*
Other^3^	226	142	(62.8)	1.59	1.13–2.22		191	51	(26.7)	1.72	1.11–2.65			
Mothers education														
≤ High school/don’t know	338	184	(54.4)	1.00		*0.46*	334	62	(18.6)	1.00		*0.074*	*0.005*	*0.32*
≥ College	268	154	(57.5)	1.13	0.82–1.56		169	43	(25.4)	1.50	0.96–2.33			
**High school**														
Study affiliation														
Academic	323	189	(52.1)	1.00		*0.029*	181	45	(24.9)	1.00		*0.11*	*0.040*	*0.010*
Vocational	244	149	(61.1)	1.44	1.04–2.01		324	61	(18.8)	0.70	0.45–1.09			
**Alcohol/drug use**														
Low	144	57	(40.4)	1.00		*< 0.001*	140	21	(15.3)	1.00		*0.003*	*< 0.001*	*0.49*
Medium	343	193	(56.3)	1.90	1.27–2.82		212	40	(18.9)	1.29	0.72–2.29			
High	118	86	(72.9)	3.96	2.34–6.71		144	45	(31.3)	2.51	1.40–4.50			
**Sexual behaviour**														
Age at first intercourse														
≥ 15 years	352	167	(47.4)	1.00		*< 0.001*	311	40	(12.9)	1.00		*< 0.001*	*< 0.001*	*0.15*
≤ 14 years	252	171	(67.9)	2.34	1.67–3.28		172	59	(34.3)	3.54	2.24–5.59			
Years sexually active														
≤ 1 year	162	54	(33.3)	1.00		*< 0.001*	171	13	(7.6)	1.00		*< 0.001*	*< 0.001*	*0.50*
≥ 2 years	442	284	(64.3)	3.60	2.46–5.26		312	86	(27.6)	4.63	2.50–8.58			
Condom use *first* intercourse														
Yes	358	198	(55.3)	1.00		*0.78*	267	40	(15.0)	1.00		*0.001*	*0.15*	*0.014*
No^4^	248	140	(56.5)	1.05	0.76–1.45		226	61	(27.0)	2.10	1.34–3.28			
Currently in relationship														
Yes	322	185	(57.5)	1.00		*0.35*	179	42	(23.5)	1.00		*0.31*	*0.001*	*0.79*
No	285	153	(53.7)	0.86	0.62–1.18		326	64	(19.6)	0.80	0.51–1.24			
Sex partners past 6 months														
0-1	352	181	(51.4)	1.00		*0.010*	273	35	(12.8)	1.00		*< 0.001*	*< 0.001*	*0.013*
≥ 2	248	154	(62.1)	1.55	1.11–2.16		194	62	(32.0)	3.19	2.01–5.09			
Life time no of sex partners														
1-2	206	64	(31.1)	1.00		*< 0.001*	218	11	(5.0)	1.00		*< 0.001*	*< 0.001*	*0.17*
3-5	191	107	(56.0)	2.83	1.87–4.26		123	31	(25.2)	6.34	3.06–13.16			
≥ 6	201	163	(81.1)	9.52	6.01–15.08		119	53	(44.5)	15.11	7.46–30.62			
**Last sexual partner**														
Age difference														
Same age or younger	144	66	(45.8)	1.00		*0.005*	413	80	(19.4)	1.00		*0.042*	*< 0.001*	*0.78*
Older (≥ 1 year)	445	264	(59.3)	1.72	1.18–2.52		54	17	(31.5)	1.91	1.03–3.57			
Condom use *last* intercourse														
Yes	94	33	(35.1)	1.00		*< 0.001*	319	76	(23.8)	1.00		*0.009*	*< 0.001*	*0.36*
No^5^	513	305	(59.5)	2.71	1.71–4.29		168	23	(13.7)	1.97	1.18–3.28			
**School based testing**														
Provision of urine sample														
Yes	564	313	(55.5)	1.00		*0.74*	470	97	(20.6)	1.00		*0.48*	*0.49*	*0.73*
No	43	25	(58.1)	1.11	0.59–2.09		35	9	(25.7)	1.33	0.60–2.93			
Chlamydia test result														
Negative	523	290	(55.4)	1.00		*0.94*	448	91	(20.3)	1.00		*0.14*	*0.11*	*0.23*
Positive	41	23	(56.1)	1.03	0.54–1.95		17	6	(35.3)	2.14	0.77–5.94			

N, total number of participants in each group; n (%), number (proportion) of participants with *clinic based testing*; OR, odds ratio; CI, confidence interval; NA, not applicable; ^1^*p*-value for equality between categories; ^2^*p-*value for interaction between gender and independent variable; ^3^Living with relatives, in students’ houses or in private accommodation; ^4^Includes the response: ‘Uncertain if any contraception was used’ (girls n = 3, boys n = 10); ^5^Includes the response: ‘Uncertain if any contraception was used’ (girls n = 3, boys n = 8).

### Chlamydia prevalence

Among participants with previous clinic based testing, chlamydia prevalence was 7.3% (95% CI 4.7–10.8) in girls and 6.2% (2.3–13.0) in boys. Among participants with school-only test, prevalence was 7.2% (4.3–11.1) in girls, and 3.0% (1.5–5.3) in boys. 50% (n = 29) of the chlamydia infected participants reported clinic based testing and 21% (n = 12) reported previous treatment. Among 41 girls with a positive chlamydia test result in the school based screening, 23 reported clinic based testing (Table 1). Among 17 boys screening positive at school, 6 reported previous clinic based testing.

### Clinic based testing

In gender-stratified crude analysis, the following variables increased the odds of clinic based testing in both girls and boys: older age, Sami/Sami-Norwegian ethnicity, residence outside the family home, higher levels of alcohol and drug use, age at first intercourse ≤ 14 years, sexual activity ≥ 2 years, ≥ 2 sexual partners past 6 months, higher number of lifetime sexual partners, age difference with last partner ≥ 1 year, and no condom use at last intercourse (Table 1). Girls in vocational classes had higher odds of clinic based testing than those with academic affiliation. No condom use at first intercourse increased the odds of clinic based testing in boys, but not in girls. Clinic based testing was not associated with school based screening or with prevalent chlamydia infection*.*

In multivariable analysis, the following variables increased the odds of clinic based testing in girls and boys combined: older age, age at first intercourse ≤ 14 years, no condom use at first intercourse, steady relationship, and having had higher number of lifetime partners (Table 2). No condom use at last intercourse increased the odds in girls only. Significant interaction was present between gender and ethnicity (*p =* 0.012) in the multivariable model. Girls had higher odds of clinic based testing than boys, but the odds ratio varied by ethnic group. Among boys, clinic based testing varied between the three ethnic groups with Norwegian boys having the lowest test activity. In girls, ethnic group was not associated with clinic based testing. Nagelkerke’s estimate of explained variance in the multivariable model for all participants was 42%.

**Table 2:** **Odds ratios for *clinic based testing *in multivariable logistic regression models**

		**Girls**			**Boys**			**All participants**^**1**^	
**Characteristic**	**OR**	**95% CI**	***p***^***2***^	**OR**	**95% CI**	***p***^***2***^	**OR**	**95% CI**	***p***^***2***^
**Gender: girls vs boys**^3^									
Norwegian	NA			NA			7.96	5.26–12.04	*< 0.001*
Sami/Sami-Norwegian	NA			NA			3.62	1.92–6.82	*< 0.001*
Other	NA			NA			1.89	0.66–5.45	*0.24*
**Age**									
OR per year	1.47	1.20–1.81	*< 0.001*	1.73	1.28–2.32	*< 0.001*	1.54	1.30–1.83	*< 0.001*
**Family and culture**									
Ethnicity girls^4^									
Norwegian	ns			NA			1.00		*0.37*
Sami/Sami-Norwegian	ns			NA			1.30	0.82–2.08	
Other	ns			NA			0.78	0.38–1.60	
Ethnicity boys^5^									
Norwegian	NA			1.00		*< 0.001*	1.00		*< 0.001*
Sami/Sami-Norwegian	NA			2.83	1.56–5.13		2.86	1.59–5.14	
Other	NA			3.53	1.44–8.64		3.28	1.37–7.84	
**Sexual behaviour**									
Age at first intercourse									
≥15 years	1.00		*0.006*	1.00		*0.003*	1.00		*< 0.001*
≤14 years	1.78	1.18–2.67		2.56	1.39–4.71		2.02	1.43–2.85	
Condom use *first* intercourse									
Yes	ns			ns			1.00		*0.013*
No^6^	ns			ns			1.48	1.09–2.01	
Currently in a relationship									
Yes	ns			ns			1.00		*0.009*
No	ns			ns			0.66	0.49–0.90	
Lifetime no of sex partners									
1-2	1.00		*< 0.001*	1.00		*< 0.001*	1.00		*< 0.001*
3-5	2.42	1.59–3.71		4.22	1.95–9.12		3.07	2.11–4.46	
≥6	6.31	3.85–10.34		8.57	3.91–18.79		7.63	5.03–11.55	
Condom use *last* intercourse									
Yes	1.00		*0.011*	ns			ns		
No^7^	1.93	1.16–3.20		ns			ns		

The significant variables from stepwise selection, age and gender (if applicable) were included in the models.

OR, odds ratio; CI, confidence interval; NA, not applicable; ns, non-significant; ^1^Interaction term between gender and ethnicity included in the model *(p 0.012)*; ^2^*p*-value for equality between categories; ^3^Reference group: boys; ^4^Odds ratios in girls; ^5^Odds ratios in boys; ^6^Includes the response: ‘Uncertain if any contraception was used’ (girls n = 3, boys n = 10); ^7^Includes the response: ‘Uncertain if any contraception was used’ (girls n = 3, boys n = 8).

### School based screening

In girls’ crude analysis, academic affiliation, condom use at first intercourse, and current urogenital symptoms increased the odds of school based screening (Additional file 1), and these variables remained significant in the girls’ multivariable model (Table 3). Among 243 girls reporting ≥ 1 symptom, only 10% had a positive test result. In boys, age at first intercourse ≥ 15 years and no prior treatment for chlamydia infection increased the odds of school based screening in crude analysis, while in multivariable analysis low substance use, age at first intercourse ≥ 15 years, and no condom use at last intercourse increased the odds. Assessing girls and boys combined, the following variables increased the odds of school based screening in multivariable analysis: male gender, academic affiliation, age at first intercourse ≥ 15 years, condom use at first intercourse, and current urogenital symptoms. Nagelkerke’s estimate in the multivariable model for all participants was 6.2% (Table 3).

**Table 3:** **Odds ratios for *school based screening *in multivariable logistic regression models including the significant variables from stepwise selection**

		**Girls**			**Boys**			**All participants**	
**Characteristic**	**OR**	**95% CI**	***p***^**1**^	**OR**	**95% CI**	***p***^**1**^	**OR**	**95% CI**	***p***^**1**^
**Gender**									
Girls^2^	NA			NA			0.57	0.34–0.97	*0.040*
**Age**									
OR per year	0.91	0.65–1.27	*0.58*	0.82	0.52–1.28	*0.39*	0.86	0.67–1.11	*0.24*
**High school**									
Study affiliation									
Academic	1.00		*0.001*	ns			1.00		*0.013*
Vocational	0.33	0.17–0.65		ns			0.51	0.30–0.87	
**Alcohol/drug use**									
Low	ns			1.00		*0.039*	ns		
Medium	ns			0.75	0.22 to 2.58		ns		
High	ns			0.26	0.08 to 0.91		ns		
**Sexual behaviour**									
Age first intercourse									
≥15 years	ns			1.00		*0.001*	1.00		*0.029*
≤14 years	ns			0.23	0.10–0.57		0.58	0.35–0.95	
Condom use *first* intercourse									
Yes	1.00		*0.010*	ns			1.00		*0.026*
No^3^	0.42	0.21–0.82		ns			0.57	0.35–0.94	
Condom use *last* intercourse									
Yes	ns			1.00		*0.003*	ns		
No^4^	ns			3.86	1.59–9.41		ns		
**Chlamydia infection**									
Urogenital symptoms^5^									
No	1.00		*<0.001*	ns			1.00		*0.001*
Yes	4.26	1.89–9.63		ns			3.23	1.57–6.65	

Age and gender (if applicable) included in the model.

OR, odds ratio; CI, confidence interval; NA, not applicable; ns non-significant, ^1^*p*-value for equality between categories; ^2^Reference group: boys; ^3^Includes the response: ‘Uncertain if any contraception was used’ (girls n = 3, boys n = 10); ^4^Includes the response: ‘Uncertain if any contraception was used’ (girls n = 3, boys n = 8); ^5^In girls: dysuri, vaginal discharge, intermenstrual and/or postcoital bleeding. In boys: dysuri and/or urethral discharge.

The 18 girls and 11 boys with chlamydia infection and school-only test reported less condom use at both first and last intercourse, and higher number of sexual partners past 6 months and during lifetime than non-infected participants with school-only test (*p <* 0.05).

## Discussion

We found that a large proportion of adolescent girls had been tested previously. The unusually high and equal participation between genders in the school based screening and the finding of a large undetected pool of chlamydia infections confirms the value of school based testing. High school based screening and clinic based testing were associated with completely different independent variables. To have been tested previously was mainly associated with traditional risk factors suggesting that these adolescents were aware of the behavioural determinants of chlamydia infection and thus were motivated by their own perceived risk. In contrast, school based screening mostly was associated with factors unknown to increase risk, strongly indicating the presence of other incentives. To our knowledge, this is the first study to compare clinic based testing to school based screening in a general adolescent population.

The high proportion of girls with clinic based testing is in agreement with Norwegian surveillance data and other European studies with young females accessing clinical test sites much more frequently than same-aged males [2,15,16]. The lower sexual activity among Norwegian boys 15–20 years and young males’ reluctance to access health care services may contribute to less testing [14,17]. The high participation rate in the school based screening may be explained by the following factors: thorough planning, the relevant topics, the universal offer to all students irrespective of sexual history, the ‘in-class’ recruitment and sampling procedures, the efficient logistics with rapid notification of positive test results, and this being the first chlamydia high school based screening in Northern Norway [18]. It is likely that invitation to participate in research increased uptake. In Norway, repeat school based studies on adolescent health and lifestyle including biological samples, have shown sustained response rates above 85% [19,20] thus suggesting a potential for sustainability of repeat school based chlamydia screening. We observed a curious and welcoming attitude among both students and staff. Male participants frequently commented on the simplicity of urinating in a cup and the convenience of a class-wise approach with everyone getting tested. Adolescent males are more likely to accept STI testing if the testing procedures are convenient [17,21,22] and if they feel that confidentiality is maintained [23]. Individual provider characteristics may also impact on their decision [24,25]. The boys’ high acceptance for school based screening challenges the notion of adolescent males as a hard-to-reach group as the selection bias normally created by low participation among boys was not observed.

### Chlamydia prevalence

High chlamydia prevalence was detected in girls irrespective of earlier test behaviour. Girls with clinic based testing had higher levels and longer duration of risk behaviours than those with school-only test. The equal infection levels may indicate effect of adherence to recommendations on testing and treatment in the first group and less adherence in the school-only test group among those with high risk behaviours. Boys with clinic based testing had approximately the same prevalence as girls. Boys with school-only test having half the prevalence of girls is consistent with less sexual activity in boys this age and thus reduced infection risk [14]. For girls and boys combined, half of chlamydia cases were detected in the school-only test group. Correspondingly these infected subjects had higher levels of risk behaviours than participants with school-only test and a negative chlamydia test result. We may have underestimated prevalence in girls as *C. trachomatis* was detected in FVU samples that are less sensitive than self-collected vaginal swabs.

### Factors associated with testing

Clinic based testing behaviour differing between ethnic groups among boys may indicate that boys’ testing patterns at this age is more influenced by same-ethnicity peers and less by national recommendations. In contrast, girls’ test activity was not associated with ethnicity. Early first intercourse doubled the odds of clinic based testing and was positively correlated with number of sexually active years suggesting that it may reflect a longer sexually active period with more testing opportunities. In contrast, participants who just recently started their sexual career only had limited time to seek chlamydia testing. No condom use at first and last intercourse increasing the odds of clinic based testing suggests testing for safety reasons. While condom use at any occasion is a dyadic behaviour and negotiable between partners, chlamydia testing can freely be carried out by the individual. Higher lifetime number of sexual partners being associated with increased clinic based testing is in agreement with a study on uptake in the English National Chlamydia Screening Programme where persons being tested in the programme reported significantly higher numbers of partners than a random sample of the general population [26].

In the multivariable model using school based screening as independent outcome, current urogenital symptoms in girls and in both genders combined were the only significant traditional risk factor. However, symptoms had low positive predictive value to detect chlamydia infection which is consistent with other studies [1]. School screening reached a large proportion of adolescents at no or low risk of chlamydia infections. Among these, participants not previously tested in clinical practice may have benefited from learning the test procedure.

One-fifth of all infections were detected in participants with previous chlamydia treatment and were thus repeat infections undetected in clinical practice. This may indicate a weakness in the Norwegian testing algorithm. Our data did not allow any conclusions about the duration of an infection, or if it was transmitted by the same partner, by new sexual contacts, or was due to treatment failure or non-compliance. In addition, we had no information on time since clinic based testing. Prevalent chlamydia infection not being associated with clinic based testing indicates that adolescents do underestimate their own infection risk as also observed in other studies [27]. Although chlamydia testing in youth clinics is easily available and free of charge irrespective of the patient meeting the national test criteria or not, our study shows that a significant proportion of adolescents at risk had not been tested before the study suggesting the presence of other barriers to testing.

The strengths of this study include the representativeness achieved by high participation, the use of computer-based questionnaires to limit social desirability bias, and the use of high quality biological samples [28].

### Limitations

The study is limited by cross-sectional design that precludes establishing causality and by self-reported data on sexual behavioural and previous chlamydia treatment. The presence of some social desirability bias is likely due to the sensitive topics. Using laboratory data to assess the outcome variable ‘clinic based testing’ instead of a questionnaire would have improved validity. However, longer recall periods have been found accurate for assessing low-frequency events such as a previous chlamydia test [29]. In this paper, we assumed specific sexual behaviours, i.e. number of sexual partners past 6 months and circumstances related to last sexual intercourse, reported before the school screening to be representative for sexual behaviours before clinic based testing. Our assumption is based on the finding that single-events like the most recent intercourse is valid representation of sexual behaviour over longer periods of time [30]. Previous STI test results could have influenced later sexual behaviour in the direction of less or increased risk causing a slight attenuation in the observed odds ratio estimates.

## Conclusions

More than half the girls had been tested prior to the school based screening and had high prevalence independent of clinic based testing. While clinic based testing was associated with traditional chlamydia risk factors, school based screening was mostly associated with factors unknown to increase infection risk. The high and equal participation between genders and the detection of a large chlamydia reservoir that included both first-time and repeat infections confirms the value of school based screening and suggests this approach to be further explored in Norway.

## Abbreviations

FVU: First-void urine; STI: Sexually transmitted infection.

## Competing interests

The authors declare that they have no competing interests.

## Authors’ contribution

KG conceived and designed the study, applied for funding, collected the data, and drafted the manuscript. GSS and ASF participated in study design. KG and TW chose the main direction for data analysis and performed the statistical analyses. All authors contributed to the interpretation of the results and revised and approved the manuscript.

## Pre-publication history

The pre-publication history for this paper can be accessed here:

http://www.biomedcentral.com/1471-2334/13/361/prepub

## Supplementary Material

Additional file 1 **Sosio-demographic and sexual behaviour characteristics – prevalence and crude odds ratios for *school based screening* in univariable logistic regression models.** Click here for file
