# Supplementary material for: Factors associated with Chlamydia trachomatis testing in a high school based screening and previously in clinical practice: a cross-sectional study in Norway
Source: BMC Infect Dis. 2013 Aug 1;13:361. doi: 10.1186/1471-2334-13-361 (PMC3751625; doi:10.1186/1471-2334-13-361)
Supplement: Additional file 1 — Sosio-demographic and sexual behaviour characteristics – prevalence and crude odds ratios for school based screening in univariable logistic regression models. [file 1471-2334-13-361-S1.doc]

**Additional file 1.** Sosio-demographic and sexual behaviour characteristics – prevalence and crude odds ratios for *school based screening* in univariable logistic regression models.

|  |  |  |  |  |  |  |  |  |  |  |  |  |  |  |  |  |  |
| --- | --- | --- | --- | --- | --- | --- | --- | --- | --- | --- | --- | --- | --- | --- | --- | --- | --- |
|  | **Girls** | | | | | |  | **Boys** | | | | | |  | **All participants** | | |
|  |  |  |  |  |  |  |  |  |  |  |  |  |  |  |  |  |  |
|  |  |  |  |  |  |  |  |  |  |  |  |  |  |  |  |  |  |
|  | **School based screening** | | | | |  |  | **School based screening** | | | | |  |  | **Interaction** | | |
|  |  |  |  |  |  |  |  |  |  |  |  |  |  |  |  |  |  |
| **Characteristic** | **N** | **n** | **(%)** | **OR** | **95% CI** | ***p***^1^ |  | **N** | **n** | **(%)** | **OR** | **95% CI** | ***p***^1^ |  | ***p***^1^ |  | ***p***^2^ |
|  |  |  |  |  |  |  |  |  |  |  |  |  |  |  |  |  |  |
|  |  |  |  |  |  |  |  |  |  |  |  |  |  |  |  |  |  |
| **Total** | 607 | 564 | (92.9) | NA |  |  |  | 505 | 470 | (93.1) | NA |  |  |  |  |  |  |
|  |  |  |  |  |  |  |  |  |  |  |  |  |  |  |  |  |  |
| **Age** |  |  |  |  |  |  |  |  |  |  |  |  |  |  |  |  |  |
| 15-16 | 179 | 168 | (93.9) | 1.00 |  | *0.78* |  | 149 | 141 | (94.6) | 1.00 |  | *0.72* |  | *0.51* |  | *1.00* |
| 17 | 193 | 177 | (91.7) | 0.72 | 0.33–1.61 |  |  | 196 | 180 | (91.8) | 0.64 | 0.27–1.53 |  |  |  |  |  |
| 18 | 177 | 166 | (93.8) | 0.99 | 0.42–2.34 |  |  | 114 | 107 | (93.9) | 0.87 | 0.31–2.47 |  |  |  |  |  |
| 19-20 | 58 | 53 | (91.4) | 0.69 | 0.23–2.09 |  |  | 46 | 42 | (91.3) | 0.60 | 0.17–2.08 |  |  |  |  |  |
|  |  |  |  |  |  |  |  |  |  |  |  |  |  |  |  |  |  |
| **Family and culture** |  |  |  |  |  |  |  |  |  |  |  |  |  |  |  |  |  |
| Ethnicity |  |  |  |  |  |  |  |  |  |  |  |  |  |  |  |  |  |
| Norwegian | 433 | 402 | (92.8) | 1.00 |  | *0.39* |  | 353 | 329 | (93.2) | 1.00 |  | *0.88* |  | *0.62* |  | *0.39* |
| Sami/Sami-Norwegian | 131 | 124 | (94.7) | 1.47 | 0.59–3.18 |  |  | 115 | 106 | (92.2) | 0.86 | 0.39–1.91 |  |  |  |  |  |
| Other | 43 | 38 | (88.4) | 0.58 | 0.22–1.60 |  |  | 36 | 34 | (94.4) | 1.24 | 0.28–5.48 |  |  |  |  |  |
|  |  |  |  |  |  |  |  |  |  |  |  |  |  |  |  |  |  |
| Residence in school year |  |  |  |  |  |  |  |  |  |  |  |  |  |  |  |  |  |
| At home | 380 | 356 | (93.7) | 1.00 |  | *0.33* |  | 314 | 290 | (92.4) | 1.00 |  | *0.42* |  | *0.86* |  | *0.15* |
| Other^3^ | 226 | 207 | (91.6) | 0.73 | 0.39–1.37 |  |  | 191 | 180 | (94.2) | 1.35 | 0.65–2.83 |  |  |  |  |  |
|  |  |  |  |  |  |  |  |  |  |  |  |  |  |  |  |  |  |
| Mothers education |  |  |  |  |  |  |  |  |  |  |  |  |  |  |  |  |  |
| < High school/don’t know | 338 | 312 | (92.3) | 1.00 |  | *0.52* |  | 334 | 308 | (92.2) | 1.00 |  | *0.31* |  | *0.26* |  | *0.69* |
| > College | 268 | 251 | (93.7) | 1.23 | 0.65–2.32 |  |  | 169 | 160 | (94.7) | 1.50 | 0.69–3.28 |  |  |  |  |  |
|  |  |  |  |  |  |  |  |  |  |  |  |  |  |  |  |  |  |
| **High school** |  |  |  |  |  |  |  |  |  |  |  |  |  |  |  |  |  |
| Study affiliaton |  |  |  |  |  |  |  |  |  |  |  |  |  |  |  |  |  |
| Academic | 323 | 347 | (95.6) | 1.00 |  | *0.002* |  | 181 | 171 | (94.5) | 1.00 |  | *0.36* |  | *0.005* |  | *0.21* |
| Vocational | 244 | 217 | (88.9) | 0.37 | 0.20–0.70 |  |  | 324 | 299 | (92.3) | 0.70 | 0.33–1.49 |  |  |  |  |  |
|  |  |  |  |  |  |  |  |  |  |  |  |  |  |  |  |  |  |
| **Alcohol/drug use** |  |  |  |  |  |  |  |  |  |  |  |  |  |  |  |  |  |
| Low | 144 | 133 | (94.3) | 1.00 |  | *0.28* |  | 140 | 132 | (96.4) | 1.00 |  | *0.11* |  | *0.046* |  | *0.71* |
| Medium | 343 | 322 | (93.9) | 0.92 | 0.40–2.13 |  |  | 212 | 200 | (94.3) | 0.63 | 0.22–1.83 |  |  |  |  |  |
| High | 118 | 106 | (89.8) | 0.53 | 0.21–1.35 |  |  | 144 | 130 | (90.3) | 0.35 | 0.12–1.01 |  |  |  |  |  |
|  |  |  |  |  |  |  |  |  |  |  |  |  |  |  |  |  |  |
| **Sexual behaviour** |  |  |  |  |  |  |  |  |  |  |  |  |  |  |  |  |  |
| Age at first intercourse |  |  |  |  |  |  |  |  |  |  |  |  |  |  |  |  |  |
| > 15 years | 352 | 330 | (93.8) | 1.00 |  | *0.33* |  | 311 | 297 | (95.5) | 1.00 |  | *0.014* |  | *0.018* |  | *0.22* |
| < 14 years | 252 | 231 | (91.7) | 0.73 | 0.39–1.37 |  |  | 172 | 154 | (89.5) | 0.40 | 0.20–0.83 |  |  |  |  |  |
|  |  |  |  |  |  |  |  |  |  |  |  |  |  |  |  |  |  |
| Years sexually active |  |  |  |  |  |  |  |  |  |  |  |  |  |  |  |  |  |
| < 1 year | 162 | 149 | (92.0) | 1.00 |  | *0.60* |  | 171 | 160 | (93.6) | 1.00 |  | *0.90* |  | *0.79* |  | *0.66* |
| > 2 years | 442 | 412 | (93.2) | 1.20 | 0.61–2.36 |  |  | 312 | 291 | (93.3) | 0.95 | 0.45–2.03 |  |  |  |  |  |
|  |  |  |  |  |  |  |  |  |  |  |  |  |  |  |  |  |  |
| Condom use *first* intercourse |  |  |  |  |  |  |  |  |  |  |  |  |  |  |  |  |  |
| Yes | 358 | 340 | (95.0) | 1.00 |  | *0.019* |  | 267 | 250 | (93.6) | 1.00 |  | *0.75* |  | *0.050* |  | *0.19* |
| No^4^ | 248 | 223 | (89.9) | 0.47 | 0.25–0.89 |  |  | 226 | 210 | (92.9) | 0.89 | 0.44–1.81 |  |  |  |  |  |
|  |  |  |  |  |  |  |  |  |  |  |  |  |  |  |  |  |  |
| Currently in a relationship |  |  |  |  |  |  |  |  |  |  |  |  |  |  |  |  |  |
| Yes | 322 | 296 | (91.9) | 1.00 |  | *0.31* |  | 179 | 169 | (94.4) | 1.00 |  | *0.38* |  | *0.84* |  | *0.19* |
| No | 285 | 268 | (94.0) | 1.39 | 0.74–2.61 |  |  | 326 | 301 | (92.3) | 0.71 | 0.33–1.52 |  |  |  |  |  |
|  |  |  |  |  |  |  |  |  |  |  |  |  |  |  |  |  |  |
| Sex partners past 6 months |  |  |  |  |  |  |  |  |  |  |  |  |  |  |  |  |  |
| 0-1 | 352 | 323 | (91.8) | 1.00 |  | *0.16* |  | 273 | 258 | (94.5) | 1.00 |  | *0.33* |  | *0.65* |  | *0.097* |
| > 2 | 248 | 235 | (94.8) | 1.62 | 0.83–3.19 |  |  | 194 | 179 | (92.3) | 0.69 | 0.33–1.46 |  |  |  |  |  |
|  |  |  |  |  |  |  |  |  |  |  |  |  |  |  |  |  |  |
| Life time no of sex partners |  |  |  |  |  |  |  |  |  |  |  |  |  |  |  |  |  |
| 1-2 | 206 | 192 | (93.2) | 1.00 |  | *0.96* |  | 218 | 206 | (94.5) | 1.00 |  | *0.19* |  | *0.38* |  | *0.49* |
| 3-5 | 191 | 178 | (93.2) | 1.00 | 0.46–2.18 |  |  | 123 | 117 | (95.1) | 1.13 | 0.42–3.11 |  |  |  |  |  |
| > 6 | 201 | 186 | (92.5) | 0.90 | 0.43–1.92 |  |  | 119 | 107 | (89.9) | 0.52 | 0.23–1.20 |  |  |  |  |  |
|  |  |  |  |  |  |  |  |  |  |  |  |  |  |  |  |  |  |
| **Last sexual partner** |  |  |  |  |  |  |  |  |  |  |  |  |  |  |  |  |  |
| Age difference |  |  |  |  |  |  |  |  |  |  |  |  |  |  |  |  |  |
| Same age or younger | 144 | 133 | (92.4) | 1.00 |  | *0.79* |  | 413 | 386 | (93.5) | 1.00 |  | *0.42* |  | *0.89* |  | *0.55* |
| Older (> 1 year) | 445 | 414 | (93.0) | 1.11 | 0.54–2.26 |  |  | 54 | 52 | (96.3) | 1.82 | 0.42–7.87 |  |  |  |  |  |
|  |  |  |  |  |  |  |  |  |  |  |  |  |  |  |  |  |  |
| Condom use *last* intercourse |  |  |  |  |  |  |  |  |  |  |  |  |  |  |  |  |  |
| Yes | 94 | 90 | (95.7) | 1.00 |  | *0.25* |  | 168 | 153 | (91.1) | 1.00 |  | *0.10* |  | *0.72* |  | *0.059* |
| No^5^ | 513 | 474 | (92.4) | 0.54 | 0.19–1.55 |  |  | 319 | 303 | (95.0) | 1.86 | 0.89–3.86 |  |  |  |  |  |
|  |  |  |  |  |  |  |  |  |  |  |  |  |  |  |  |  |  |
| **Chlamydia infection** |  |  |  |  |  |  |  |  |  |  |  |  |  |  |  |  |  |
| Clinic based testing |  |  |  |  |  |  |  |  |  |  |  |  |  |  |  |  |  |
| No | 269 | 251 | (93.3) | 1.00 |  | *0.74* |  | 399 | 373 | (93.5) | 1.00 |  | *0.48* |  | *0.49* |  | *0.73* |
| Yes | 338 | 313 | (92.6) | 0.90 | 0.48–1.68 |  |  | 106 | 97 | (91.5) | 0.75 | 0.34–1.66 |  |  |  |  |  |
|  |  |  |  |  |  |  |  |  |  |  |  |  |  |  |  |  |  |
| Previous treatment |  |  |  |  |  |  |  |  |  |  |  |  |  |  |  |  |  |
| No | 485 | 452 | (93.2) | 1.00 |  | *0.59* |  | 467 | 438 | (93.8) | 1.00 |  | *0.027* |  | *0.11* |  | *0.16* |
| Yes | 122 | 112 | (91.8) | 0.82 | 0.39–1.71 |  |  | 37 | 31 | (83.8) | 0.34 | 0.13–0.89 |  |  |  |  |  |
|  |  |  |  |  |  |  |  |  |  |  |  |  |  |  |  |  |  |
| Urogenital symptoms^6^ |  |  |  |  |  |  |  |  |  |  |  |  |  |  |  |  |  |
| No | 351 | 317 | (90.3) | 1.00 |  | *0.003* |  | 460 | 429 | (93.3) | 1.00 |  | *0.82* |  | *0.011* |  | *0.24* |
| Yes | 251 | 243 | (96.8) | 3.26 | 1.48–7.17 |  |  | 35 | 33 | (94.3) | 1.19 | 0.27–5.20 |  |  |  |  |  |
|  |  |  |  |  |  |  |  |  |  |  |  |  |  |  |  |  |  |

N, total number of participants in each group; n (%), number (proportion) of participants with *school based screening*; OR, odds ratio; CI, confidence interval;

NA: not applicable; ^1^*p*-value for equality between categories; ^2^*p-*value for interaction between gender and variable; ^3^Living with relatives, in students’ houses

or in private accommodation; ^4^Includes the response: ‘Uncertain if any contraception was used’ (girls n=3, boys n=10); ^5^Includes the response: ‘Uncertain if

any contraception was used’ (girls n=3, boys n=8); ^6^In girls: dysuri, vaginal fluor, intermenstrual and/or postcoital bleeding. In boys: dysuri and/or urethral

discharge
